# Age-Related Variation in Foraging Behaviour in the Wandering Albatross at South Georgia: No Evidence for Senescence

**DOI:** 10.1371/journal.pone.0116415

**Published:** 2015-01-09

**Authors:** Hannah Froy, Sue Lewis, Paulo Catry, Charles M. Bishop, Isaac P. Forster, Akira Fukuda, Hiroyoshi Higuchi, Ben Phalan, Jose C. Xavier, Daniel H. Nussey, Richard A. Phillips

**Affiliations:** 1 Institute of Evolutionary Biology, University of Edinburgh, Edinburgh, United Kingdom; 2 British Antarctic Survey, Natural Environment Research Council, Cambridge, United Kingdom; 3 Marine and Environmental Sciences Centre, ISPA—Instituto Universitário, Lisbon, Portugal; 4 School of Biological Sciences, Bangor University, Bangor, United Kingdom; 5 Australian Antarctic Division, University of Tasmania, Kingston, Australia; 6 Shizuoka University, Shizuoka, Japan; 7 Keio University, Fujisawa, Japan; 8 Department of Zoology, University of Cambridge, Cambridge, United Kingdom; 9 Marine and Environmental Sciences Centre, University of Coimbra, Coimbra, Portuga; Norwegian Polar Institute, NORWAY

## Abstract

Age-related variation in demographic rates is now widely documented in wild vertebrate systems, and has significant consequences for population and evolutionary dynamics. However, the mechanisms underpinning such variation, particularly in later life, are less well understood. Foraging efficiency is a key determinant of fitness, with implications for individual life history trade-offs. A variety of faculties known to decline in old age, such as muscular function and visual acuity, are likely to influence foraging performance. We examine age-related variation in the foraging behaviour of a long-lived, wide-ranging oceanic seabird, the wandering albatross *Diomedea exulans*. Using miniaturised tracking technologies, we compared foraging trip characteristics of birds breeding at Bird Island, South Georgia. Based on movement and immersion data collected during the incubation phase of a single breeding season, and from extensive tracking data collected in previous years from different stages of the breeding cycle, we found limited evidence for age-related variation in commonly reported trip parameters, and failed to detect signs of senescent decline. Our results contrast with the limited number of past studies that have examined foraging behaviour in later life, since these have documented changes in performance consistent with senescence. This highlights the importance of studies across different wild animal populations to gain a broader perspective on the processes driving variation in ageing rates.

## Introduction

Age-related variation in demographic parameters is widely documented among long-lived, iteroparous species [1–3]. This variation in survival and reproductive rates has major consequences for individual fitness, and important implications for evolutionary and ecological dynamics [4–5]. Survival probability and reproductive performance are often observed to increase with age, and this is generally attributed to the advantages of increased experience, particularly during early life [6–7]. In later life, senescence, or the decline in physiological function that accompanies increasing adult age, can result in higher mortality rates and poor reproductive success of older individuals [8]. Although there is now abundant evidence for age-related changes in survival and reproduction in wild vertebrate populations [9], the mechanisms driving these important demographic changes are still not fully understood [10].

Individuals differ in their ability to exploit prey in the surrounding environment, and since this determines the resources an individual has available to allocate between reproduction and maintenance, variation in foraging performance is likely to be a key source of demographic variation [11]. Despite the challenges of studying behaviour in wild systems, improvements in foraging in early life have been documented in several species [6, 12–13]. As well as being subject to developmental constraints (e.g. [14]), young or inexperienced animals may choose inappropriate habitats or patches in which to forage; they may be poor at searching for or recognising suitable prey items; and they may be less skilled at capturing prey than more experienced foragers [6]. Foraging ability may therefore increase with age, which will contribute to age-related variation in demographic rates. These advancements may occur during the juvenile and immature phases, as individuals develop motor skills and improve their knowledge of the environment [15–17]. They may also occur in mature adults; in particular if breeding imposes new constraints on foraging behaviour, animals may improve as they gain further experience [18–19].

Despite having gained a reasonable understanding of the beneficial effects of experience on foraging efficiency in young and middle-aged individuals, we still know very little about age-related changes in foraging behaviour that may occur in later life [20]. Although studying senescence in wild populations is challenging [21], there is now evidence from a range of taxa that demographic senescence has wide-ranging implications [10]. A wealth of research on laboratory organisms and humans has demonstrated age-related declines in a wide variety of relevant traits, such as activity levels [22], visual acuity [23], and athletic ability [24]. In addition, an increasing number of wild animal studies have also recorded senescent declines in traits such as body mass [25], immune function [26], and muscle composition [27]. Since many of these factors are likely to impact foraging ability, declines in foraging performance may be a key driver of reduced survival and fecundity in older vertebrates. A handful of studies have examined how foraging behaviour changes in potentially senescent wild animals [20, 28–33]. However, our knowledge of age-related variation in foraging behaviour in later life is still very limited, as is our understanding of its causes and the consequences for senescent decline.

Birds are relatively long-lived in comparison to mammals, and seabirds in particular are among the longest-lived vertebrates [34]. This longevity makes seabirds useful models for the study of age-related variation in the natural environment [35], particularly since they are colonial breeders and exhibit high site fidelity, which makes them amenable to monitoring [36]. Longitudinal studies over many years have provided convincing evidence of age-related changes in demographic rates during both early and later life in a number of species [37–40]. However, the study of foraging behaviour is more challenging since this occurs at sea, and many seabirds travel great distances to find food in remote areas. The development of small tracking devices means it is now possible to collect information on the behaviour of birds on distant foraging trips [41]. Research using these technologies has revealed increases in flight or foraging efficiency with age in juveniles and immatures [16–17], and also with increasing breeding experience in mature birds [19], which likely contribute to changes in demographic rates with age. A limited number of studies have also explored age-related variation in foraging behaviour in later life, finding evidence for changes in activity levels [20], trip duration and efficiency [28, 30], and general foraging strategy [31].

Here, we examine age-related variation in foraging behaviour in the wandering albatross *Diomedea exulans*. Like all seabirds, wandering albatrosses are central-place foragers during the breeding season that must return to the colony to attend their nests. Our study examined foraging behaviour in birds breeding at Bird Island, South Georgia, using two related datasets. The first involved a targeted tracking study to compare the behaviour of young and old birds during the incubation period of the 2012 breeding season. Males and females take turns incubating the egg, and must endure long periods of fasting while their partners are foraging at sea [42]. Thus, birds are constrained during the incubation period and under strong selection to forage efficiently to avoid nest desertion. The second involved a larger dataset, combining tracking data collected between 1991 and 2012 during different stages of the breeding cycle. This population shows extensive age-related variation in reproduction, with first time breeders performing poorly compared to experienced breeders, and individuals showing signs of reproductive senescence from the mid- to late-teens [43]. We hypothesised that this variation in reproductive performance may be driven by age-related variation in foraging behaviour, potentially involving an interaction with sex given the high degree of sexual size dimorphism and evidence for spatial segregation in this species during the breeding season [44–45].

In particular, we hypothesised that first time breeders may have reduced foraging competency, and may therefore have a higher take-off and landing rate if they are less skilled at recognising and capturing prey [6]. An earlier study of wandering albatrosses found that inexperienced birds foraged more actively at night [46], and therefore we expected that higher take-off and landing rates would be particularly evident during darkness. We also hypothesised that first time breeders might compensate for reduced foraging efficiency by taking longer foraging trips than young birds with more breeding experience. We hypothesised that older individuals would show signs of senescence similar to those documented in other seabirds. Based on previous studies, we expected older individuals to exhibit lower take-off and landing rates than younger birds [20], and to spend more time resting on the water [30]. Finally, we hypothesised that older birds would take longer [28] or more distant foraging trips [30], and tested for differences in foraging distribution by comparing latitudes and longitudes of the most distal points of the foraging trip [30].

## Materials and Methods

Fieldwork was carried out at a wandering albatross breeding colony on Bird Island, South Georgia (54°00’S, 38°03’W), which is the subject of a long-term monitoring study by the British Antarctic Survey. We first tested for effects of age, experience and sex on foraging parameters derived from the 2012 incubation study, before expanding our analysis to test for age-related variation in foraging parameters that could be reliably and consistently derived from tracking data collected at all stages of the breeding season since 1991.

### Ethics statement

Handling time during the deployment and retrieval of logging devices was <10 minutes, and the total device weight was ~0.5% of adult body weight, which is well below the recommended upper threshold limit [47]. The average breeding success of tracked birds did not differ from the population mean. All fieldwork was approved by the British Antarctic Survey Ethics Committee and carried out under permit from the Govt. of South Georgia and the South Sandwich Islands.

### 2012 incubation study

**Figure 1:**
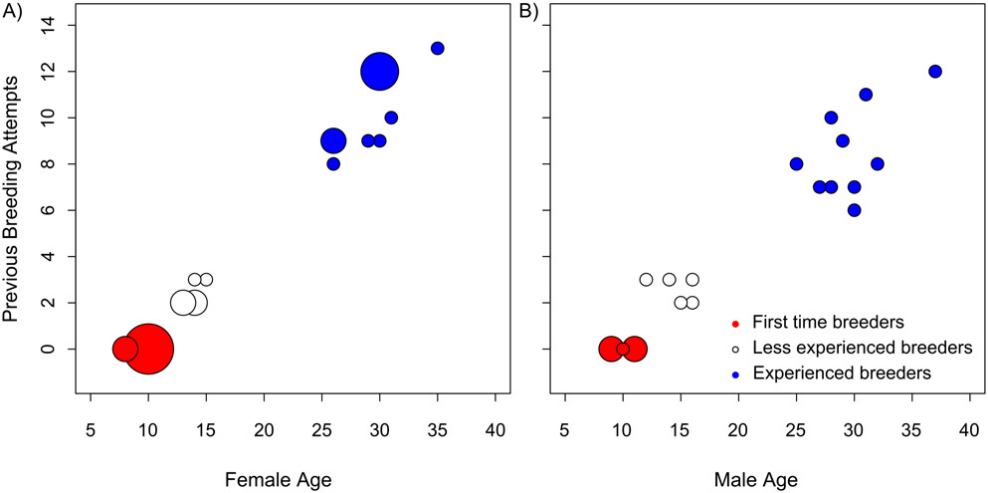
The age and breeding experience of wandering albatrosses tracked during the incubation period in 2012. A) Females n = 22; B) Males n = 20. The size of the circles indicates the number of individuals (n = 1–4) with each combination of age and breeding experience. The colour of the circles indicates the breeding experience class used in the analysis.

Wandering albatrosses were tracked for a single foraging trip during the incubation period of the 2012 breeding season (Jan-Feb 2012). Tracking devices were deployed on 42 birds of known age and breeding experience: 22 females and 20 males (Fig. 1A & 1B, respectively). To test for age-related variation in foraging behaviour, we targeted young birds aged between 8 and 16 years (n = 22; Fig. 1, red and white circles), and old birds aged between 25 and 37 years (n = 20; Fig. 1, blue circles). All old birds were experienced breeders, having bred on 6–13 previous occasions. Half of the young birds tracked were first time breeders (n = 11; Fig. 1, red circles), and the remainder were less experienced breeders that had bred on two or three previous occasions (n = 11; Fig. 1, white circles).

Each bird was fitted with two different loggers, which provided concurrent information on location and activity patterns for the duration of the foraging trip. IgotU GPS loggers (GT-120, manufactured by Mobile Action Technology) were sealed in heat-shrink tubing and attached to the mantle feathers with Tesa tape. They recorded location (latitude and longitude, accurate to ~15 m) at 25 minute sampling intervals. Birds were also fitted with combined Global Location Sensing—immersion loggers (MK19; British Antarctic Survey, Cambridge), which were attached to plastic leg rings with cable ties. These devices have a salt-water sensor which detects immersion in seawater, providing information on activity patterns throughout the foraging trip. Time spent dry indicates the bird is in flight, whereas wet time represents foraging activity or sitting on the water. The immersion devices recorded the timing of any transitions between wet and dry states (to the nearest 3 seconds, providing the change of state lasts ≥6 seconds).

All analyses were performed using the statistical program R (version 2.15.2). To identify erroneous GPS locations, fixes that indicated flight speeds over 90 km.h^-1^ were visually examined [48]. Those that did not fit the flight trajectory were deemed inaccurate and excluded (this represented only two GPS fixes). The last GPS fix in the colony prior to departure and the first GPS fix after arrival back in the colony were used to define the start and end of the foraging trip. GPS data were unavailable for two birds because the device was lost before they returned to the colony, and activity data were unavailable for two birds because the immersion logger failed to download. Five GPS tracks were incomplete because the battery failed during the trip. In these cases, the activity data were used to define the end of the foraging trip where possible, with the final transition from a wet to dry state used to indicate approximate arrival back at the colony. These incomplete tracks were deemed ‘near-complete’ if the flight trajectory and the remaining trip duration indicated that the birds were on their return trip, having apparently reached the furthest point from the colony, or point of inflection (see S1 Fig.).

For each complete or near-complete foraging trip (n = 38), we calculated the following parameters: (i) trip duration (in days); (ii) maximum distance from colony (in km, distance from the colony at the point of inflection, calculated using function *spDistsN1* in package *sp*); (iii) bearing at the point of inflection (in degrees, calculated using function *earth.bear* in package *fossil*); (iv) latitude and (v) longitude at the point of inflection.

For each complete foraging trip (n = 35), we also calculated: (vi) trip distance (in km, sum of point-to-point distances between GPS fixes, calculated using function *deg.dist* in package *fossil*); (vii) mean speed (in km.h^-1^, trip distance divided by trip duration). All calculations used great-circle distances, accounting for the curvature of the earth.

The activity data (n = 39) were used to calculate: (viii) landing rate (wet events per hour, calculated as the total number of wet-dry transitions during the trip divided by the trip duration in hours); (ix) landing rate for bouts on the water lasting ≥12 seconds (as landing rate, but the number of wet events was reduced by ~25%); (x) wet time (proportion of total trip spent wet).

The GPS tracks were interpolated at 10 minute intervals using function *redisltraj* in package *adehabitatLT*[49]. The function *crepuscule* in package *maptools* was then used to determine the time of civil twilight (when the sun is 6 degrees below the horizon) and assign each GPS fix to daylight (including twilight) or darkness; hereafter day or night. The mean speed was then calculated for both day and night separately (in km.h^-1^, point-to-point distance divided by time interval between fixes). Day and night were also assigned to activity fixes by rounding the date-time of each wet-dry transition to the nearest 10 minutes, and then matching with the corresponding GPS fix for that track. These were used to calculate the landing rate and wet time (as above) for both day and night. Since wandering albatrosses are primarily diurnal feeders [50–51], we expect landings and wet time during daylight to be predominantly driven by foraging activity, whereas wet time during the night is more likely to represent time spent resting on the water.

The relationships between foraging trip characteristics, and age and breeding experience were then examined using generalised linear models. Our first model tested for the effect of age with a two-level factor for age class (young, 8–16 years; or old, 25–37 years). Individuals in the old age category were of an age at which reproductive senescence is likely to be manifest [43]. Because age and breeding experience are correlated (Fig. 1), we were unable to fully resolve the effect of both factors. However, previous work on this population indicates that the majority of improvement in breeding success in early life reflects the lower success of first time breeders, whereas individuals with at least one previous breeding attempt have similar average reproductive success [43]. Therefore, our second model examined the effect of breeding experience using a two-level factor for whether or not it was an individual’s first breeding attempt. Finally, to test whether both age and breeding experience were important, our third model included a three-level factor for breeding experience (first time; less experienced, 2–3 previous attempts; or experienced, 6 or more previous attempts).

Sex differences in foraging trip characteristics were tested with a two-level factor for sex. We ran one model with sex alone, and also three models that included sex and each of the factors for age and experience in an additive manner. Finally, to test whether the age or experience effects differed between the sexes, we ran models including an interaction between sex and each of the three age and experience factors outlined above.

These models were compared (ten in total, plus a null model) using Akaike Information Criterion (AIC). Whilst the best model is taken to be that with the lowest AIC value, AIC differences of less than two are not considered to be meaningfully different [52]. Therefore, if the inclusion of an additional factor did not improve the AIC by two or more, then the more parsimonious model (with fewer terms) was accepted as the best model [53]. Wet time was modelled as a proportion, using a quasibinomial error distribution since the data were overdispersed. All other variables were modelled with a Gaussian error distribution.

### 1991–2012 combined tracking study

Wandering albatrosses of known age and breeding experience were also tracked between 1991 and 2009 for other studies [45, 51, 54]. Location data were collected using either ARGOS-Platform Terminal Transmitters or GPS loggers, with different sampling regimes employed in different years (see S1 Table). Combined with the 2012 data, this represents data on the foraging trips of 207 breeding birds tracked during different stages of the breeding cycle: incubation (n = 56); brood guard (n = 58); and post-brood chick-rearing (n = 93). For each foraging trip, we calculated: trip duration; maximum distance from colony; bearing at the point of inflection; and latitude and longitude at the point of inflection, as above. Trip distances and speeds could not be compared readily among foraging trips because of varying intervals between fixes resulting from changes in Argos satellite coverage and differences in GPS sampling regimes. As above, we included all complete or near-complete tracks in the analysis. To avoid pseudoreplication, we included only the first complete foraging trip from each bird.

We tested for age-related variation in the characteristics of all foraging trips from 1991 to 2012 using generalised additive models (GAMs), which allow for non-linear relationships. This was preferred to the factorial approach outlined above, because it took advantage of the more even distribution of ages in the larger dataset, and did not require assumptions about the form of the relationships. Analyses were performed in package *mgcv* [55], with a smoother spline for age, and constraining the degrees of freedom to a maximum of 5 to avoid an overly complex age function. Data from birds aged >35 were pooled to avoid any biases resulting from extreme values associated with small sample sizes. The model included a two-level factor for sex, and an interaction between sex and the age function to test for sex-specific effects. We also tested a three-level factor for breeding stage (incubation; brood guard; or post-brood), and accounted for annual variation in environmental conditions by including year as a factor. This also accounted for variation associated with the different devices or sampling regimes. We could not test independently for an effect of breeding experience because only 16 out of the 207 tracked birds were first time breeders, resulting in an unbalanced two-level factor. AIC model selection was used to assess the significance of each of the terms in the model, as above. Unless indicated otherwise, data are given as the mean ± standard error.

## Results

### 2012 incubation study

**Figure 2:**
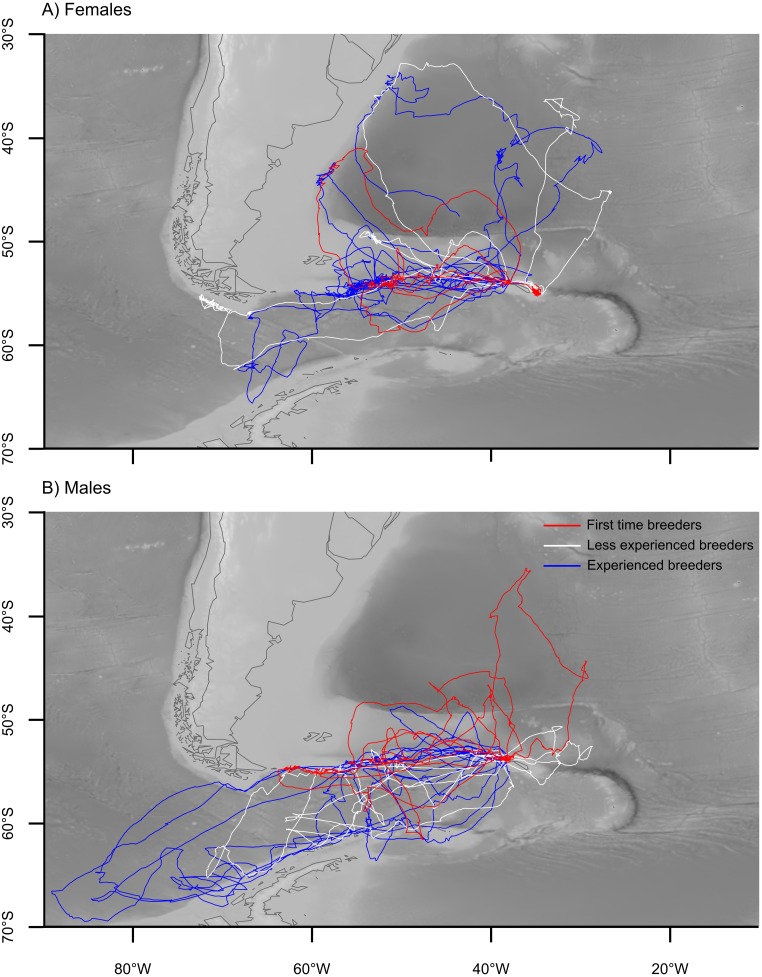
The foraging trips of wandering albatrosses tracked with GPS loggers during the incubation period in 2012. A) Females n = 19; B) Males n = 20. The background indicates bathymetry (ocean depth).

Wandering albatrosses breeding at Bird Island foraged over a large area during the incubation period of 2012, ranging from 33–70°S and 7–90°W (Fig. 2). Foraging trips lasted an average of 13 days, but ranged between 4 and 24 days. On average, birds travelled 6027.2 ± 410.4 km on a foraging trip, reaching 1578.6 ± 112.6 km from the colony. The mean travel speed was 20.18 ± 0.79 km.h^-1^, and birds travelled faster during the day than the night (day: 24.59 ± 0.74 km.h^-1^; night: 10.20 ± 0.95 km.h^-1^; t(64) = 11.95, p<0.001). On average, birds made 1.79 ± 0.20 landings per hour, with the rate of landings for wet bouts lasting 12 seconds or more 1.61 ± 0.08 per hour. The rate was slightly higher during the day than during the night, but this was not statistically significant (day: 1.86 ± 0.11 per hour; night 1.63 ± 0.11 per hour; t(72) = 1.49, p = 0.140). Birds spent around half of the time away from the colony on the water (proportion of foraging trip: 0.50 ± 0.01), and a larger proportion of their time wet during the night (0.62 ± 0.02) than during the day (0.44 ± 0.02; t(72) = 7.10, p<0.001).

**Figure 3:**
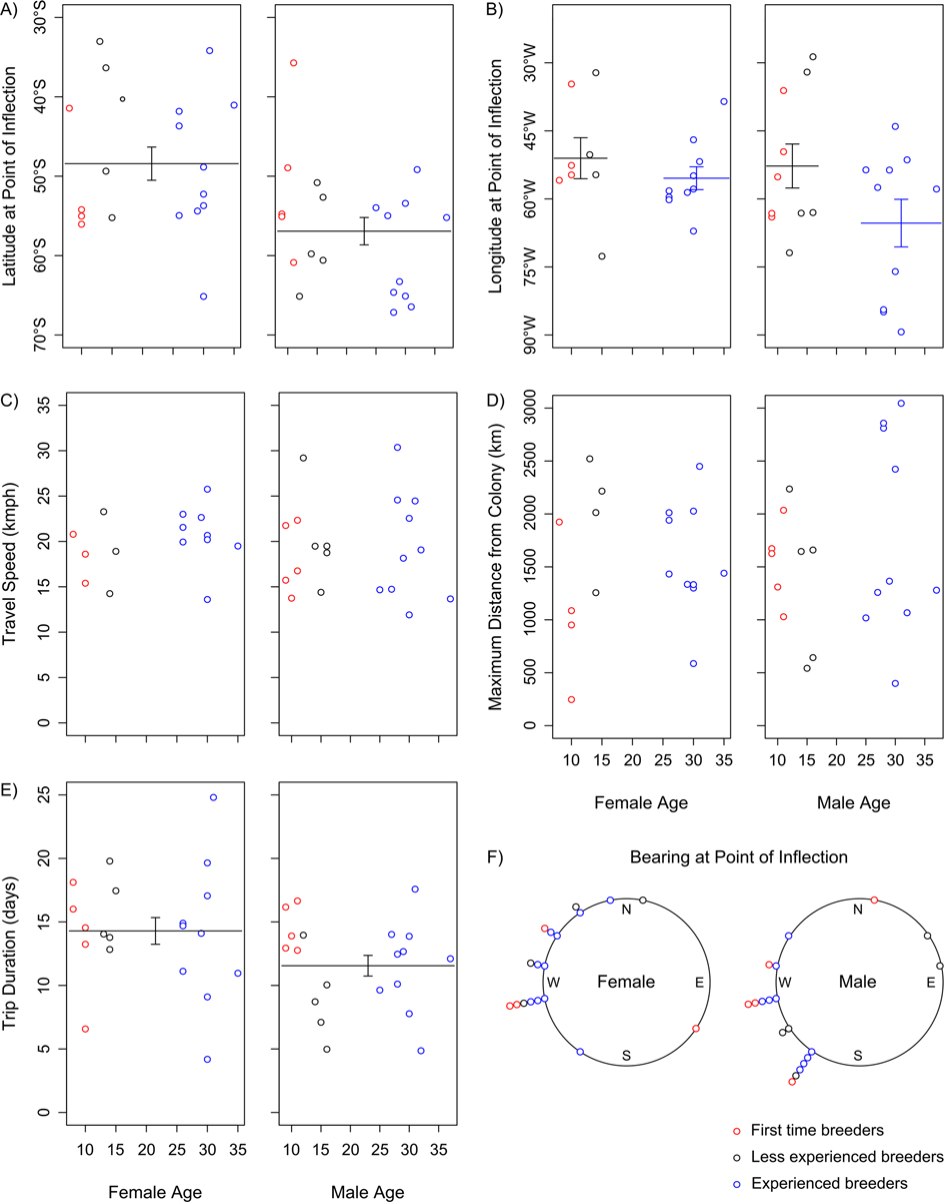
Incubation foraging trip characteristics for birds of different age and breeding experience in 2012. A) Latitude; B) Longitude; C) Travel speed; D) Maximum distance from colony; E) Trip duration; F) Bearing. Points show raw data, and lines indicate differences in average trait values (± standard error) for either age or breeding experience classes indicated by the best model (although not all are included in the most parsimonious model, see Table 1 and Results).

There was no significant effect of sex, age or breeding experience on distance travelled, maximum distance from the colony, or mean travel speed (Fig. 3C & 3D; see Table 1 for results of model selection). Males undertook foraging trips of slightly shorter mean duration (males: 11.61 ± 0.81 days; females: 14.35 ± 1.05 days; Fig. 3E; Table 1; see S2 Table for full models) and also travelled further south than females (males: 56.89°S ± 1.73; females: 48.37°S ± 2.09; Fig. 3A; Table 1). The models suggested these sex differences may be driven by the males with some breeding experience (Fig. 3A & 3E), but models including an interaction between breeding experience and sex were within ∆AIC<2 of the model including only sex (Table 1). There was some evidence that older birds travelled further west (young: 51.92°W ± 3.27; old: 60.32°W ± 3.05; Fig. 3B), but the model including age class was within ∆AIC<2 of the more parsimonious null model (Table 1). These differences in latitude and longitude were reflected in the bearing at the point of inflection; older birds tended to have travelled in a more south-westerly direction (young: -55.64° ± 18.62; old: -94.29° ± 8.67; Fig. 3F), but again, the model including age class was within ∆AIC<2 of the null model (Table 1).

**Table 1:** Model selection tables examining the effect of age and breeding experience on characteristics of wandering albatross foraging trips, birds tracked during the incubation period of 2012.

	**∆AIC compared to best model**								
	**Trip duration**	**Trip distance**	**Mean speed**	**Max dist from col**	**Bearing**	**Inflection latitude**	**Inflection longitude**	**Day ptp speed**	**Night ptp speed**
Sample size	40	35	35	38	38	38	38	35	35
Age Class * Sex	3.88	4.57	4.94	4.88	1.86	2.56	1.08	4.88	3.52
First Breeding Attempt * Sex	0.39	3.21	4.64	2.98	5.47	*0.00*	4.58	4.75	3.36
Breeding Experience * Sex	2.42	6.69	8.08	4.48	4.93	2.32	4.93	7.19	6.66
Age Class + Sex	1.90	2.61	3.41	3.21	*0.00*	1.02	0.02	3.50	2.09
First Breeding Attempt + Sex	1.00	2.66	2.67	2.23	3.71	2.03	2.62	2.80	1.95
Breeding Experience + Sex	2.90	4.51	4.66	4.22	1.80	3.02	2.02	4.59	3.36
Sex	***0.00***	0.67	1.89	1.97	2.47	**0.60**	2.04	1.52	2.46
Age Class	4.15	1.88	1.48	1.26	**1.06**	9.03	***0.00***	1.96	0.17
First Breeding Attempt	3.34	2.00	0.74	0.28	4.23	9.59	2.29	1.22	***0.00***
Breeding Experience	5.24	3.78	2.73	2.26	2.87	11.02	2.00	3.06	1.44
Null	2.23	***0.00***	***0.00***	***0.00***	2.86	7.92	**1.56**	***0.00***	**0.47**

The best model in each case is highlighted in italics (where ∆AIC = 0). The most parsimonious model in each case is highlighted in bold (where ∆AIC<2 compared to the best model).

**Figure 4:**
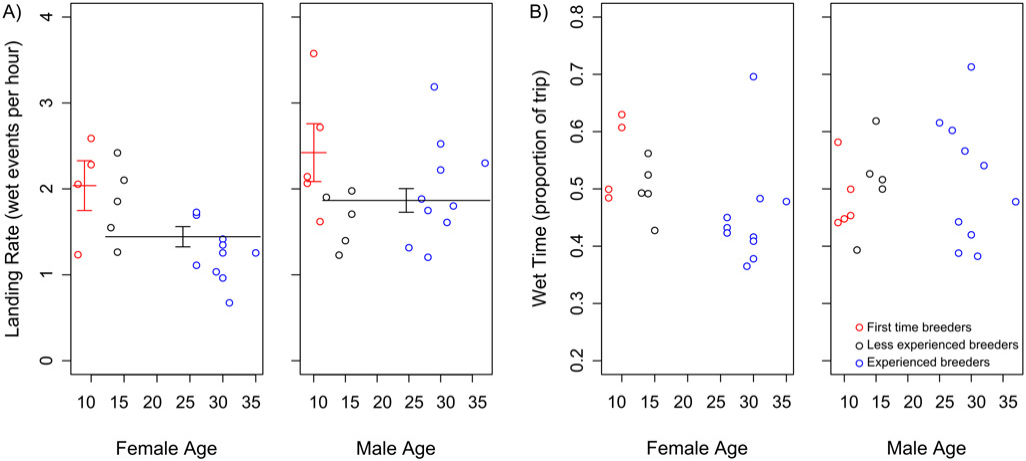
Incubation foraging trip activity patterns for birds of different age and breeding experience in 2012. A) Landing rate; B) Wet time. Points show raw data, and lines indicate differences in average trait values (± standard error) for either age or breeding experience classes indicated by the best model (although not all are included in the most parsimonious model, see Table 2 and Results).

Males landed more frequently on the water than females (males: 2.01 ± 0.14 wet-dry transitions per hour; females: 1.57 ± 0.12 per hour; Fig. 4A; see Table 2 for results of model selection). First time breeders also landed more frequently than those with previous breeding experience (first time breeders: 2.25 ± 0.22 per hour; birds with experience: 1.66 ± 0.10 per hour; Fig. 4A). The rate of landings for wet bouts lasting ≥12 seconds showed the same patterns (Table 2). During the day, the landing rate showed the same pattern as the overall rate, with males and first time breeders landing more often (males: 2.07 ± 0.15 wet-dry transitions per hour; females: 1.66 ± 0.15 per hour; first time breeders: 2.50 ± 0.27 per hour; birds with previous breeding experience: 1.69 ± 0.10 per hour; Table 2). However, during the night only the sex effect was apparent (males: 1.93 ± 0.16 wet-dry transitions per hour; females: 1.27 ± 0.11 per hour; Table 2). Sex, age or breeding experience were not included in the most parsimonious models for the proportion of time spent on the water during a foraging trip, either overall, or during day or night (Fig. 4B; Table 2).

**Table 2:** Model selection tables examining the effect of age and breeding experience on activity characteristics of wandering albatross foraging trips, birds tracked during the incubation period of 2012.

	**∆AIC compared to best model**						
	**Landing rate**	**Landing rate >12s**	**Wet time**	**Landing rate day**	**Wet time** **day**	**Landing rate night**	**Wet time night**
Sample size	39	39	39	37	37	37	37
Age Class * Sex	2.69	3.89	0.52	7.14	2.12	2.55	*0.00*
First Breeding Attempt * Sex	2.39	3.31	1.87	1.44	1.47	3.24	1.01
Breeding Experience * Sex	*0.00*	*0.00*	3.70	3.40	4.94	*0.00*	2.02
Age Class + Sex	4.15	5.53	1.35	6.81	1.24	2.77	4.79
First Breeding Attempt + Sex	**0.40**	**1.31**	2.26	***0.00***	1.05	2.28	5.75
Breeding Experience + Sex	1.99	2.72	3.22	1.91	2.83	4.22	6.62
Sex	6.27	8.56	1.66	8.83	0.36	**0.82**	3.81
Age Class	7.89	8.12	*0.00*	9.27	0.67	10.60	2.80
First Breeding Attempt	4.30	4.03	0.71	2.82	0.19	9.99	3.78
Breeding Experience	5.95	5.51	1.78	4.68	2.00	11.98	4.66
Null	9.68	10.84	**0.35**	11.55	***0.00***	8.81	**1.88**

The best model in each case is highlighted in italics (where ∆AIC = 0). The most parsimonious model in each case is highlighted in bold (where ∆AIC<2 compared to the best model).

### 1991–2012 combined dataset

**Figure 5:**
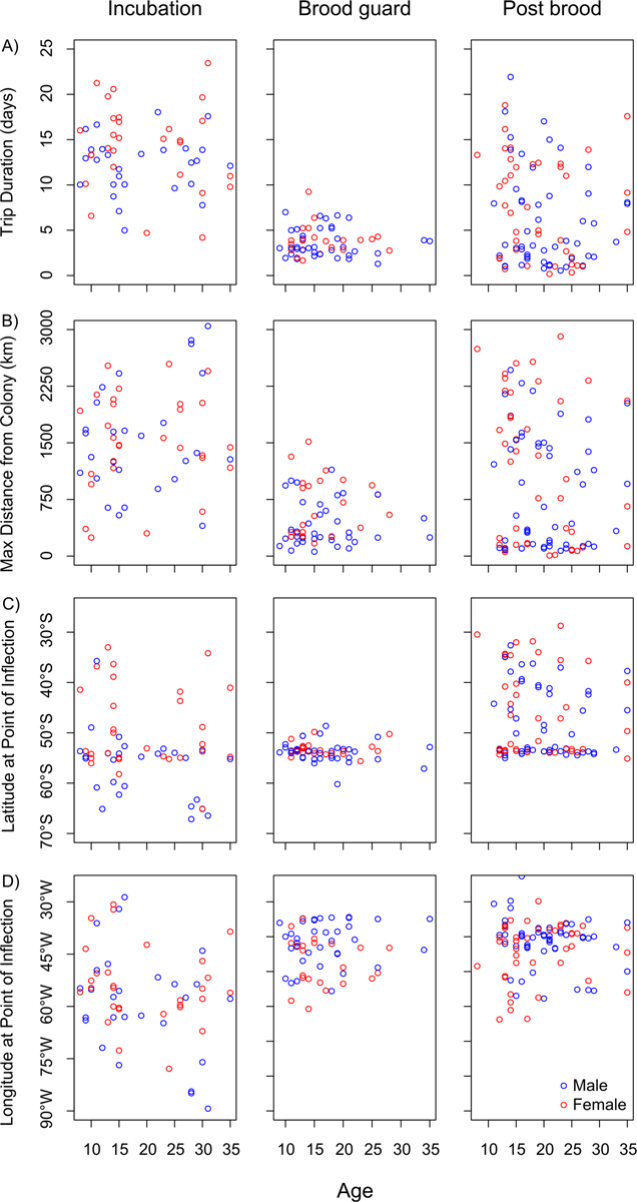
Characteristics of foraging trips for birds of different age tracked between 1991 and 2012. A) Trip duration; B) Maximum distance from colony; C) Latitude; D) Longitude. Points show raw data.

There was little evidence for age-related variation in the analysis of foraging trip characteristics of all available tracking data. An age term was not included in the most parsimonious models for trip duration, or distance from the colony, bearing, latitude or longitude at the point of inflection (Fig. 5; see Table 3 for results of model selection). Trip duration varied among the different stages of the breeding cycle, foraging trips during incubation were longest, followed by those in post-brood, and then brood guard (incubation: 13.27 ± 0.55 days; brood guard: 3.75 ± 0.21 days; post-brood: 6.91 ± 0.65 days; Fig. 5A; Table 3; see S3 Table for full models). Males also undertook trips of shorter duration on average than females (males: 6.76 ± 0.48 days; females: 8.95 ± 0.70 days; Fig. 5A; Table 3). These differences were also reflected in the distance from the colony at the point of inflection; incubation and post-brood trips were the furthest (inc: 1535.0 ± 90.1 km; brood: 519.9 ± 47.0 km; post-brood: 991.7 ± 89.7 km; Fig. 5B; Table 3), and males did not travel as far as females (males: 882.5 ± 70.6 km; females: 1158.5 ± 85.4 km; Fig. 5B; Table 3). Birds foraged further north on post-brood trips than during other stages of the breeding cycle (inc: 52.77°S ± 1.11; brood: 53.60°S ± 0.24; post-brood: 47.22°S ± 0.81; Fig. 5C; Table 3), and also further west during the incubation period (inc: 56.53°W ± 1.78; brood: 43.76°W ± 0.93; post-brood: 42.69°W ± 0.81; Fig. 5D; Table 3). Males also travelled further south on average than females (males: 52.00°S ± 0.63; females: 48.68°S ± 0.82; Fig. 5C; Table 3).

**Table 3:** Model selection tables examining the effect of age and breeding experience on characteristics of wandering albatross foraging trips, breeding birds tracked between 1991 and 2012.

	**∆AIC compared to best model**				
	**Trip duration**	**Max dist from col**	**Bearing**	**Inflection latitude**	**Inflection longitude**
Sample size	207	207	207	207	207
Age * Sex + Year + Stage	0.51	3.12	2.01	1.51	4.62
Age * Sex + Year	45.64	18.74	10.26	17.53	32.19
Age * Sex + Stage	14.40	2.72	15.57	3.67	0.47
Age * Sex	100.29	48.84	31.51	41.13	60.33
Age + Sex + Year + Stage	0.99	2.24	2.20	*0.00*	5.07
Age + Sex + Stage	12.48	1.86	16.60	1.94	0.84
Age + Sex + Year	44.72	17.73	11.22	16.81	32.03
Age + Stage + Year	5.42	5.15	0.38	12.60	3.64
Sex + Stage + Year	***0.00***	0.32	1.75	**0.72**	4.63
Year + Stage	4.58	3.28	***0.00***	13.50	3.14
Sex + Stage	13.72	***0.00***	15.39	4.27	1.00
Sex + Year	43.02	15.73	10.24	15.54	30.62
Age + Stage	14.84	3.97	14.64	11.30	*0.00*
Age + Year	49.51	21.17	9.26	26.79	31.28
Age + Sex	97.15	47.47	30.62	39.14	58.75
Age	101.12	50.73	28.84	47.49	58.20
Sex	101.04	46.69	29.39	38.16	66.38
Year	47.91	19.19	8.30	25.70	29.80
Stage	16.66	2.19	13.41	14.28	**0.02**
Null	106.02	50.97	27.58	46.65	66.52

The best model in each case is highlighted in italics (where ∆AIC = 0). The most parsimonious model in each case is highlighted in bold (where ∆AIC<2 compared to the best model).

## Discussion

In this study, there was very little evidence for age-related variation in the foraging behaviour of wandering albatrosses breeding at Bird Island, South Georgia. Although there was an observed effect of sex, and to some extent breeding experience, there was no detectable sign of senescent decline in foraging performance in older birds. This contrasts with the small number of previous, comparable studies on seabirds that found significant effects of age on foraging in later life (see below). Here, we first discuss the lack of age-related variation in wandering albatrosses at South Georgia in the context of this previous research, and go on to discuss the effects we did detect, those of sex and breeding experience.

We found no evidence of age-related variation in the duration of foraging trips, the distance travelled, or the maximum distance reached from the colony during incubation by wandering albatrosses at South Georgia (Figs. 3D & 3E). By contrast, old male grey-headed albatrosses breeding at Bird Island took longer foraging trips than younger males during incubation [28], and old male wandering albatrosses breeding at Crozet in the south Indian Ocean travelled greater overall distances and had longer foraging ranges [30, 56]. Given that older birds also showed reduced breeding success, it was hypothesised that the age-related changes in trip parameters were indicative of senescent decline. Older birds also gained less mass per day at sea [28], and showed smaller reductions in stress hormone levels [30], lending support to the senescence hypothesis. Although these studies included larger numbers of older birds, reproductive senescence is evident at earlier ages in the wandering albatrosses breeding at Bird Island [43], and we would expect reproductive senescence to be apparent in our sample of birds aged over 25 years. However, we failed to detect the signs of senescence in foraging performance that might be expected to underpin such declines in reproductive performance (given previous results [28, 30, 56]). This is despite similar sample sizes and study design. Expanding our analysis to include data from birds tracked in all stages of the breeding cycle and in different years greatly increased our sample size, but we were still unable to detect any age-related variation (Figs. 5A & 5B).

Lecomte et al. [30] documented striking spatial segregation in foraging distribution between young and old male wandering albatrosses during the incubation period; only males over 30 years old foraged in Antarctic waters. Our data did not indicate there was spatial segregation between old and young birds of either sex at South Georgia, either during the incubation period (Figs. 3A & 3B), or at other stages of the breeding cycle (Figs. 5C & 5D). These breeding colonies are located in different oceans, with distinctive oceanographic influences, and likely very different constraints on foraging [54, 57]. Indeed, wandering albatrosses rearing chicks at Crozet adopt a dual foraging strategy, alternating long and short foraging trips [44], whereas no significant bimodality in frequency distributions of trip durations is evident at South Georgia [54]. This suggests a major difference in foraging strategy between the two regions in response to the relative availability of feeding habitat, diet or prey biogeography [54]. It may therefore be that changes in foraging behaviour in old birds are more difficult to detect in wandering albatrosses at South Georgia given the prevailing environmental conditions.

The selective forces that influence the wandering albatross populations in the south Indian and Atlantic oceans have also differed over recent decades [58–59]. These differences relate largely to the high spatial and temporal variability in fishing effort and presumed bird bycatch rates in the different regions [60]. If individuals are consistent in their foraging locations, then mortality in fisheries may result in the selective removal of subsets of the population. This selective disappearance may have differential effects on different cohorts of birds if there are long-term changes in fishing pressures, which could result in associations between age and foraging location. Longitudinal studies involving the tracking of birds on multiple foraging trips over many years would be necessary to separate these effects, and are essential if we want to investigate the within-individual changes in foraging performance that may underpin demographic senescence.

We also failed to detect any difference in landing rates, or other aspects of activity at sea, between old and young birds. Differences in activity levels have been documented in other studies using very similar methods. Old Cory’s shearwaters, *Calonectris diomedea*, took-off and landed at lower rates than mid-aged males in foraging trips during incubation [20] and, like wandering albatrosses at Crozet [30], old males spent more time sitting on the water [20]. However, it is important to note that these variables can be difficult to interpret. Because taking off from the water is energetically expensive, these results may indicate that older birds are less physically fit, and may therefore be an indicator of senescent decline. Alternatively, fewer take-offs and landings could reflect increased efficiency in experienced foragers, i.e., these birds are less likely to make unsuccessful attempts at prey capture [20]. In contrast with both these studies, we found no difference between young and old birds in the proportion of the trip spent sitting on the water, overall or during the day or night (Fig. 4B; Table 2). However, data on the body condition of tracked birds would be valuable as this could provide information on foraging efficiency. Research on the northern elephant seal *Mirounga angustirostris* has suggested that older individuals may be able to compensate for senescent declines in physiological function through the benefits of increased experience [32]. It may be that senescent birds pay a price for maintaining their foraging behaviour, for instance losing body mass, or delivering less food to their offspring.

We did observe a higher landing rate in first time breeders, both males and females (Fig. 4A). Bearing in mind the issue of interpretation outline above, this result could reflect inefficient foraging, with inexperienced birds attempting to capture prey under unsuitable conditions, failing to recognise appropriate prey items, or not having sufficient skill to successfully manipulate and secure individual items [6]. Because taking off from the water is energetically expensive for albatrosses, this higher activity rate could contribute to the observed lower breeding success of first time breeders [43]. However, without data on the success rates of individual foraging bouts (e.g. from stomach temperature probes), or information on daily mass gains during trips, we are unable to verify this hypothesis. Interestingly, the higher rate of landing on the water was only apparent in first time breeders during daylight, not during the night (Table 2). This is inconsistent with previous studies of wandering albatross in the Indian Ocean which found that inexperienced birds foraged more at night than experienced breeders, which was thought to compensate for their lower foraging efficiency [46]. This difference could relate to the relative profitability of particular foraging strategies (e.g. in-flight searching during daylight vs. sit-and-waiting on the sea surface at night), in the two regions.

We found that male wandering albatrosses travelled significantly further south on foraging trips, both during incubation in 2012 (Figs. 2 & 3A), and on average when considering all stages of the breeding cycle (Fig. 5C). This is consistent with earlier studies of this population [45, 61], and of wandering albatrosses elsewhere [44, 62]. This is thought to be a product of the larger body size, and higher wing loading of males, which makes them better adapted to the high winds at more southerly latitudes [63]. We found that males at South Georgia generally undertook shorter foraging trips throughout the breeding season and did not travel as far from the colony (Figs. 3E, 5A & 5B). Males were also more active on the water during both daylight and darkness, with a higher landing rate compared to females (Fig. 4A). Given the degree of spatial segregation between the sexes (Figs. 2 & 3A), it is plausible that the higher landing rates observed in males could reflect a higher rate of prey encounter. This is supported by previous studies that recorded higher rates of daily mass gain in males at sea during incubation [42], and a greater overall contribution to food delivery to chicks [64–65]. However, as the capture rate or energetic content of individual items may differ among habitats and therefore between sexes, ancillary data on profitability would be required to support this hypothesis.

To conclude, we found very limited evidence in this study for age-related variation in foraging behaviour in later life. This emphasises that despite technological advances, there are still many challenges associated with revealing the mechanisms underpinning demographic senescence in wild animal populations. In particular, there are difficulties in interpreting cross-sectional data, and hence longitudinal studies which examine within-individual changes in behaviour over a lifetime can be more revealing. Although the sample sizes in our series of targeted deployments during incubation were modest, the number of individuals was comparable to previous studies that have documented striking effects of age. Additional physiological data would have been extremely valuable, enabling us to examine body condition and foraging efficiency, since senescent birds may be paying a price for maintaining their foraging behaviour that we were unable to detect in this analysis. Nonetheless, our results raise important questions about the degree of consistency among breeding populations subject to varying selective pressures in the surrounding environment, which could provide insight into the mechanisms acting to shape age-related variation in different systems [25]. By comparing different populations of the same or closely related species, it should be possible to identify the interaction between intrinsic and extrinsic drivers of variation in the ageing process.

## Supporting Information

S1 Fig An example of a wandering albatross GPS track. The device was deployed for a single foraging trip during the incubation period in 2012. This track was deemed ‘near-complete’ based on the remaining trip duration, and because the bird appears to be on the return trip to the colony (red circle) having passed the point of inflection (blue circle). Trip metrics relating to the point of inflection are therefore still informative, but total trip distance and speed estimates are likely to be biased and so were not calculated.
(DOCX)

S1 Table Wandering albatrosses tracked during different stages of the breeding cycle in given years. Table shows device types and samples sizes for the combined tracking study. The sampling interval indicates the sampling regime used for the GPS devices, or average fix interval for the Platform Terminal Transmitter (PTT) devices.
(DOCX)

S2 Table The relationships between wandering albatross foraging trip characteristics and age, sex and breeding experience in 2012. Table shows the most parsimonious models as determined by model selection (see Tables 1 & 2).
(DOCX)

S3 Table The relationships between wandering albatross foraging trip characteristics and age, sex, breeding stage and year in 1991–2012. Table shows the most parsimonious models as determined by model selection (see Table 3). Only years that differed significantly are reported.
(DOCX)
