# Supplementary material for: Age-Related Variation in Foraging Behaviour in the Wandering Albatross at South Georgia: No Evidence for Senescence
Source: PLoS One. 2015 Jan 9;10(1):e0116415. doi: 10.1371/journal.pone.0116415 (PMC4289070; doi:10.1371/journal.pone.0116415)
Supplement: S1 Fig — The device was deployed for a single foraging trip during the incubation period in 2012. This track was deemed ‘near-complete’ based on the remaining trip duration, and because the bird appears to be on the return trip to the colony (red circle) having passed the point of inflection (blue circle). Trip metrics relating to the point of inflection are therefore still informative, but total trip distance and speed estimates are likely to be biased and so were not calculated. (DOCX) [file pone.0116415.s001.docx]

**Figure S1. An example of a wandering albatross GPS track.**


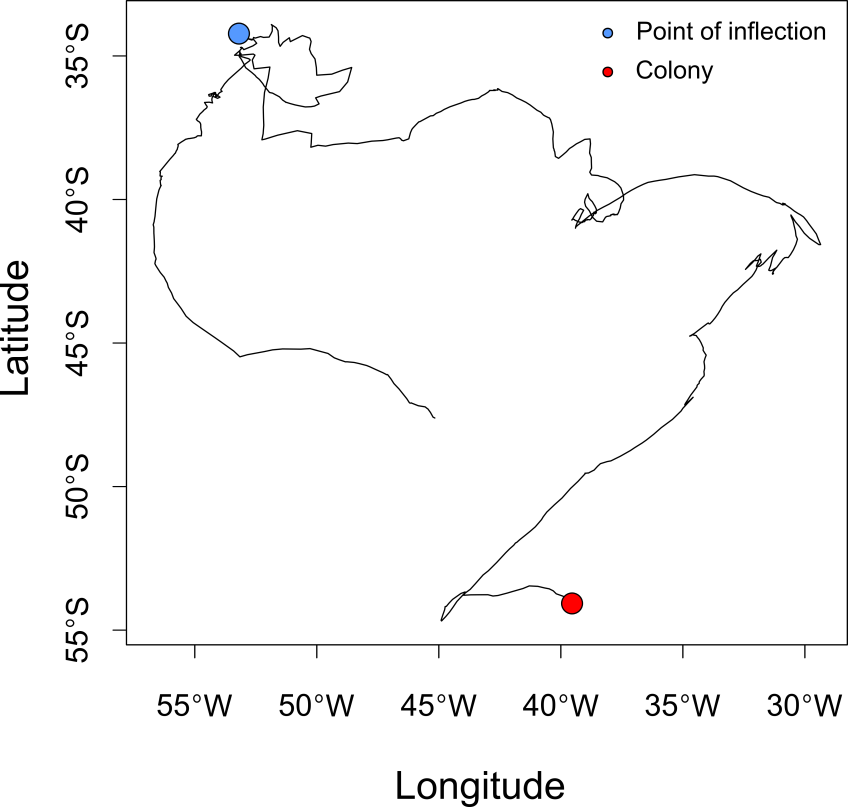


The device was deployed for a single foraging trip during the incubation period in 2012. This track was deemed ‘near-complete’ based on the remaining trip duration, and because the bird appears to be on the return trip to the colony (red circle) having passed the point of inflection (blue circle). Trip metrics relating to the point of inflection are therefore still informative, but total trip distance and speed estimates are likely to be biased and so were not calculated.
