# Supplementary material for: Age-Related Variation in Foraging Behaviour in the Wandering Albatross at South Georgia: No Evidence for Senescence
Source: PLoS One. 2015 Jan 9;10(1):e0116415. doi: 10.1371/journal.pone.0116415 (PMC4289070; doi:10.1371/journal.pone.0116415)
Supplement: S1 Table — Table shows device types and samples sizes for the combined tracking study. The sampling interval indicates the sampling regime used for the GPS devices, or average fix interval for the Platform Terminal Transmitter (PTT) devices. (DOCX) [file pone.0116415.s002.docx]

**Table S1. Wandering albatrosses tracked during different stages of the breeding cycle in given years.**

| Year | Device type | Sampling interval (mins.) | Incubation | Brood guard | Post-brood |
| --- | --- | --- | --- | --- | --- |
| 1991 | PTT | 100 | - | 5 | 2 |
| 1992 | PTT | 100 | 4 | 2 | - |
| 1998 | PTT | 180 | 6 | 11 | 3 |
| 1999 | PTT | 210 | - | - | 13 |
| 2000 | PTT | 120 | - | 8 | 11 |
| 2002 | PTT | 60 | - | - | 27 |
| 2003 | GPS | 15 | - | 23 | - |
| 2004 | GPS | 30, 60, 120 | 10 | 9 | 16 |
| 2009 | GPS | 20 | - | - | 21 |
| 2012 | GPS | 25 | 36 | - | - |

Table shows device types and samples sizes for the combined tracking study. The sampling interval indicates the sampling regime used for the GPS devices, or average fix interval for the Platform Terminal Transmitter (PTT) devices.
