# Supplementary material for: Age-Related Variation in Foraging Behaviour in the Wandering Albatross at South Georgia: No Evidence for Senescence
Source: PLoS One. 2015 Jan 9;10(1):e0116415. doi: 10.1371/journal.pone.0116415 (PMC4289070; doi:10.1371/journal.pone.0116415)
Supplement: S2 Table — Table shows the most parsimonious models as determined by model selection (see Tables 1 & 2). (DOCX) [file pone.0116415.s003.docx]

**Table S2. The relationships between wandering albatross foraging trip characteristics and age, sex and breeding experience in 2012.**

|  |  | Estimate | Std Error | p-value |  |
| --- | --- | --- | --- | --- | --- |
| Trip duration | Intercept | 14.345 | 0.938 | <0.001 | *** |
|  | Sex (males) | -2.733 | 1.327 | 0.046 | * |
| Bearing | Intercept | -55.64 | 14.41 | <0.001 | *** |
|  | Age Class (old) | -38.65 | 19.87 | 0.060 | . |
| Inflection latitude | Intercept | -48.373 | 1.952 | <0.001 | *** |
|  | Sex (males) | -8.512 | 2.691 | 0.003 | ** |
| Inflection longitude | Intercept | -51.920 | 3.242 | <0.001 | *** |
|  | Age Class (old) | -8.398 | 4.468 | 0.068 | . |
| Landing rate | Intercept | 1.449 | 0.129 | <0.001 | *** |
|  | First BA | 0. 574 | 0. 202 | 0.007 | ** |
|  | Sex (males) | 0.414 | 0.171 | 0.020 | * |
| Wet time | Intercept | 0.012 | 0.070 | 0.861 |  |
|  | Age Class (old) | -0.150 | 0.099 | 0.135 |  |
| Landing rate day | Intercept | 1.476 | 0.142 | <0.001 | *** |
|  | First BA | 0.767 | 0.226 | 0.002 | ** |
|  | Sex (males) | 0.406 | 0.186 | 0.037 | * |
| Landing rate night | Intercept | 1.274 | 0.146 | <0.001 | *** |
|  | Sex (males) | 0.653 | 0.198 | 0.002 | ** |

Table shows the most parsimonious models as determined by model selection (see Tables 1 & 2).
