# Supplementary material for: Age-Related Variation in Foraging Behaviour in the Wandering Albatross at South Georgia: No Evidence for Senescence
Source: PLoS One. 2015 Jan 9;10(1):e0116415. doi: 10.1371/journal.pone.0116415 (PMC4289070; doi:10.1371/journal.pone.0116415)
Supplement: S3 Table — Table shows the most parsimonious models as determined by model selection (see Table 3). Only years that differed significantly are reported. (DOCX) [file pone.0116415.s004.docx]

**Table S3. The relationships between wandering albatross foraging trip characteristics and age, sex, breeding stage and year in 1991 – 2012**.

|  |  | Estimate | Std Error | p-value |  |
| --- | --- | --- | --- | --- | --- |
| Trip Duration | Intercept | 6.841 | 1.778 | <0.001 | *** |
|  | Sex (M) | -1.609 | 0.643 | 0.013 | * |
|  | Stage (I) | 9.572 | 1.370 | <0.001 | *** |
|  | Stage (PB) | 3.867 | 1.171 | 0.001 | ** |
|  | Year (2002) | -4.281 | 2.095 | 0.042 | * |
|  | Year (2009) | -5.766 | 2.145 | 0.008 | ** |
| Max dist from col | Intercept | 644.05 | 110.17 | <0.001 | *** |
|  | Sex (M) | -200.03 | 98.19 | 0.043 | * |
|  | Stage (I) | 987.34 | 131.65 | <0.001 | *** |
|  | Stage (PB) | 457.32 | 117.16 | <0.001 | *** |
| Bearing | Intercept | -45.833 | 23.550 | 0.053 | . |
|  | Stage (I) | -16.017 | 18.483 | 0.387 |  |
|  | Stage (PB) | 44.123 | 15.823 | 0.006 | ** |
|  | Year (2003) | 53.730 | 26.780 | 0.046 | * |
|  | Year (2009) | -52.687 | 28.982 | 0.071 | . |
| Inflection latitude | Intercept | -48.612 | 2.551 | <0.001 | *** |
|  | Sex (M) | -3.494 | 0.922 | <0.001 | *** |
|  | Stage (I) | 0.805 | 1.965 | 0.682 |  |
|  | Stage (PB) | 6.936 | 1.680 | <0.001 | *** |
|  | Year (2009) | -7.187 | 3.077 | 0.021 | * |
| Inflection longitude | Intercept | -43.760 | 1.242 | <0.001 | *** |
|  | Stage (I) | -12.767 | 1.772 | <0.001 | *** |
|  | Stage (PB) | 1.074 | 1.583 | 0.498 |  |

Table shows the most parsimonious models as determined by model selection (see Table 3). Only years that differed significantly are reported.
